# First Case of Bioterrorism-Related Inhalational Anthrax in the United States, Palm Beach County, Florida, 2001

**DOI:** 10.3201/eid0810.020354

**Published:** 2002-10

**Authors:** Marc S. Traeger, Steven T. Wiersma, Nancy E. Rosenstein, Jean M. Malecki, Colin W. Shepard, Pratima L. Raghunathan, Segaran P. Pillai, Tanja Popovic, Conrad P. Quinn, Richard F. Meyer, Sharif R. Zaki, Savita Kumar, Sherrie M. Bruce, James J. Sejvar, Peter M. Dull, Bruce C. Tierney, Joshua D. Jones, Bradley A. Perkins, Florida Investigation Team

**Affiliations:** * Centers for Disease Control and Prevention, Atlanta, Georgia, USA; † Florida Department of Health, Tallahassee, Florida, USA; ‡ Palm Beach County Department of Public Health, West Palm Beach, Florida, USA; § Florida Department of Health, Miami, Florida, USA

**Keywords:** Anthrax, Bacillus anthracis, bioterrorism, nasal swab cultures, environmental cultures

## Abstract

On October 4, 2001, we confirmed the first bioterrorism-related anthrax case identified in the United States in a resident of Palm Beach County, Florida. Epidemiologic investigation indicated that exposure occurred at the workplace through intentionally contaminated mail. One additional case of inhalational anthrax was identified from the index patient’s workplace. Among 1,076 nasal cultures performed to assess exposure, Bacillus anthracis was isolated from a co-worker later confirmed as being infected, as well as from an asymptomatic mail-handler in the same workplace. Environmental cultures for B. anthracis showed contamination at the workplace and six county postal facilities. Environmental and nasal swab cultures were useful epidemiologic tools that helped direct the investigation towards the infection source and transmission vehicle. We identified 1,114 persons at risk and offered antimicrobial prophylaxis.

In Florida, human anthrax has been rare; among eight human cases reported in Florida in the 20th century, the most recent was a cutaneous case in 1974 (1). On October 2, 2001, a 63-year-old Florida man was hospitalized for a nonlocalizing severe illness that began 2 days earlier, characterized by fever, chills, sweats, fatigue, and malaise, which progressed to vomiting, confusion, and incoherent speech. No history of cough, dyspnea, abdominal pain, diarrhea, or skin lesions was reported. On October 4, the Florida Department of Health (FDOH) Bureau of Laboratories confirmed B. anthracis from a culture of cerebrospinal fluid. The patient’s condition deteriorated, and he died 3 days after admission (2).

After anthrax was confirmed and in consideration of possible bioterrorism, we initiated an investigation to determine the extent and source of the event, develop control strategies, and protect potentially exposed persons. This report summarizes the findings of our epidemiologic investigation.

## Methods

### Case Investigation

We performed a detailed investigation of the index patient’s exposures during the 60 days before his illness. We visually inspected and obtained culture specimens for Bacillus anthracis at locations he visited during the 60-day period, including his home, recreational destinations, retail outlets patronized, and workplace. Initial samples from the workplace were from the patient’s work area and the company mailroom and photo library, as well as air ventilation filters.

### Case-Finding and Surveillance

A confirmed case of anthrax was defined as a clinically compatible cutaneous, inhalational, or gastrointestinal illness confirmed as anthrax by laboratory tests, including 1) isolation of B. anthracis from an affected tissue or site or 2) other laboratory evidence of B. anthracis infection based on at least two supportive laboratory tests (3). Supportive laboratory tests included polymerase chain reaction (PCR) (4) of DNA from patient fluid from a normally sterile site, immunohistochemical staining of patient tissue samples, and enzyme-linked immunosorbent assay serologic tests to detect immunoglobulin G (IgG) response to B. anthracis protective antigen (PA) (5).

We implemented case-finding through daily chart review in Palm Beach County intensive-care units (ICUs) and regionally in ICUs in North Carolina, where the index patient had traveled during the potential exposure period. ICU patients who had blood or cerebrospinal fluid cultures performed within 24 hours of hospital admission had more detailed chart reviews and interviews. If anthrax was not ruled out, further interviews were done with patients, family members, and medical providers. Laboratory testing for B. anthracis and other potentially causative pathogens was offered if indicated. Nearby counties implemented similar case-finding efforts in ICUs and emergency departments.

We initiated enhanced surveillance locally through alerts to medical examiners and statewide through requests to laboratory directors to forward to the FDOH laboratories any cultures suspicious for Bacillus species isolated from sterile sites. A statewide veterinary alert was issued for cases of anthrax in animals. All case-finding surveillance was retrospective to September 11, 2001, and prospective beginning October 5.

### Surveillance in Potentially Exposed Groups

Workplace-exposed persons were defined as those who, within 60 days of onset of illness in the index patient, spent >1 h in the building where he worked. On October 3 through the employer, on October 8 through press releases and media briefings, and on October 8–10,13, 17, and 19 through information bulletins, we asked workplace-exposed persons and medical personnel caring for them to report influenzalike illness or skin lesions to the FDOH. Beginning October 8, hospitals were notified through infection-control professionals and public health alerts.

We obtained nasal swabs from workplace-exposed persons while dispensing prophylactic antibiotics on October 8–10 and from workers who handled trash at the workplace on October 13. Immediately after specimens were obtained, nasal swabs were applied to sheep-blood agar culture medium plates and transported to the Florida Public Health Laboratory. B. anthracis was confirmed in nonmotile, nonhemolytic isolates by gamma-phage lysis and PCR and later by detection of B. anthracis capsule and cell-wall antigens with direct fluorescent antibody tests. Testing for serum IgG antibody response to the PA component of the anthrax toxins was offered on October 10, 13, 17, and 19 to workplace-exposed persons.

We conducted interviews to investigate contaminated mail as an anthrax transmission vehicle at the workplace and to estimate incubation periods among anthrax patients. Persons who reported seeing or handling mail perceived as unusual or suspicious and persons with suspected anthrax exposure based on nasal swab cultures or preliminary serologic test results were interviewed to describe details surrounding unusual mail incidents as well as their routine exposure to the mail.

On October 12, we obtained nasal swabs from postal workers most likely to have handled contaminated mail at two county postal facilities that supplied mail to the workplace. We initiated anthrax surveillance on October 25 among postal employees in Palm Beach County through postal worker illness reports, a toll-free hotline for postal employees, and hospital infection-control professional reports of postal worker hospitalizations in Palm Beach County.

### Environmental Investigation

We collected bulk objects (e.g., filters from heating, ventilation, and air conditioning [HVAC] units, mail, soil samples) and swab, wipe, vacuum, and air specimens to test for B. anthracis environmental contamination by standard collection and shipping techniques (6). Control samples were routinely performed.

After contamination was confirmed at the workplace, we performed focused environmental sampling on October 8–10. Samples were obtained at work areas of the index patient and persons identified with potential B. anthracis exposures through nasal swab cultures, preliminary serologic test results, and interviews. Samples were also obtained from trash receptacles, items removed from the building, and the company mail van. Subsequent sampling throughout the 68,000 square-foot, three-story building was performed on October 25–November 8, 2001, to characterize the extent of contamination in the workplace. Samples were obtained from all floors, the parking garage, and the roof.

Beginning on October 12, 2001, we obtained surface samples for cultures at Palm Beach County postal facilities that processed workplace mail. We obtained samples from mail facilities sequentially, in reverse order of a route the mail most likely followed to arrive at the workplace. Facilities from postal routes serving two workplace buildings were tested. One route included three postal facilities that process >99% of mail the workplace received, and another route included four other postal facilities that might process workplace mail if the mail had been sent to a previous office, vacated by the company 13 months earlier. We sampled areas in each facility where workplace mail was most likely to have been processed.

Selected environmental and clinical specimen isolates of B. anthracis were analyzed by determining base-pair sequences in designated portions of isolate DNA to characterize subtypes, and sequences were then compared. Base sequence analysis was performed by multiple-locus variable-number of tandem repeat typing analysis (MLVA) techniques (7).

### Prophylaxis and Control Measures

We offered prophylactic antibiotics for B. anthracis to workplace-exposed persons (8). Until the risk for Florida postal workers could be assessed, we initiated prophylaxis for selected postal workers most likely to have handled workplace mail at two local postal facilities. Subsequent adjunct vaccination was later made available for workplace-exposed persons (9).

## Results

### Case Investigation

An autopsy of the index patient supported the diagnosis of inhalational anthrax. Autopsy findings included markedly enlarged hemorrhagic mediastinal lymph nodes on gross examination and laboratory detection of B. anthracis by immunohistochemical tests in mediastinal lymph nodes, spleen, liver sinusoids, and phagocytic cells.

The patient had no reported exposure typically associated with naturally occurring anthrax, including exposure to animals or animal products potentially harboring B. anthracis spores. He worked as a photo editor for a national media company that produces tabloid newspapers and other publications. He bicycled and fished for recreation, and his only travel in the 60 days before symptom onset on September 30 was a 5-day automobile trip to North Carolina. No typical naturally occurring anthrax sources were seen at any location inspected, and no B. anthracis contamination was detected among 44 samples from nonworkplace specimens. B. anthracis was identified in 2 of 12 specimens obtained on October 5: from the index patient’s computer keyboard and his mailbox in the company mailroom.

Workplace interviews regarding mail exposure showed that the index patient rarely handled or opened workplace mail, but co-workers recalled that he had examined a piece of stationery containing a fine, white, talc-like powder on September 19. The patient was observed holding the stationery close to his face as he looked at it over his computer keyboard.

### Case-Finding and Surveillance

No anthrax cases were detected in Palm Beach County ICU patients, although six patients underwent extensive follow-up from >500 medical charts reviewed through October 31, 2001. No anthrax cases were reported through surveillance by medical examiners. An autopsy was performed to rule out anthrax in one case reported through surveillance of medical examiners and Palm Beach County ICUs, and the patient was determined not to have anthrax. Through 2001, FDOH laboratories reported no B. anthracis isolations among 293 clinical isolates received to rule out anthrax. No reports of veterinary anthrax were received through the Florida Department of Agriculture and Consumer Services. No anthrax cases were reported through nearby county case-finding efforts among persons not exposed in the workplace.

### Surveillance among Potentially Exposed Groups

Among six workplace-exposed persons who were extensively evaluated after medical providers reported their illness, one was also identified among Palm Beach ICU patients, and inhalational anthrax was confirmed in another, a Miami-Dade County resident. This second case-patient was a 73-year-old mail distributor and co-worker of the index patient, who was reported by his medical provider on October 4. His illness began on September 28, and he was admitted to a hospital in Miami-Dade County on October 1, 2001. A nasal swab culture obtained on October 5 showed B. anthracis, but cultures from blood, bronchial washings, and pleural fluid, obtained after initiation of antibiotics, were negative. Two specimens of pleural fluid obtained on October 5 and 12 were tested by PCR and were positive for B. anthracis. Immunohistochemical staining of B. anthracis capsule and cell-wall antigens from pleural fluid cytology preparations and from transbronchial and pleural biopsy tissues obtained on October 5 and 12 were positive. Serial serum samples, obtained on October 7, 10, 11, and 17, indicated a serum IgG antibody response to the PA component of the anthrax toxin consistent with acute B. anthracis infection. The patient was treated with antibiotics and was discharged from the hospital on October 17 (10).

Of 1,076 nasal cultures obtained from workplace-exposed persons, two yielded B. anthracis. The first was the second case-patient, and the second was from an asymptomatic mail sorter in the same workplace. Nasal swab cultures obtained from two workers that handled workplace trash did not yield B. anthracis.

Interviews with employees regarding suspicious mail showed that the two workplace-exposed persons with nasal cultures positive for B. anthracis had extensive mail exposure. One, the second case-patient, was the workplace mail distributor; he did not generally open mail and did not recall handling or seeing any mail containing powder or described as unusual or as hate mail. He picked up 10,000–15,000 pieces of mail from the post office each weekday in the company mail van and distributed it at the workplace. The other co-worker, a 36-year-old woman, sorted mail and opened mail addressed to a periodical different from the one to which the index patient contributed. She recalled opening an envelope that released powder in her office on or about September 25. Afterwards, she discarded it in the trash without reading it. The letter most likely had arrived during the previous 2 weeks while she was on vacation. No other workplace mail likely to contain B. anthracis was suggested through further interviews.

**Figure 1:**
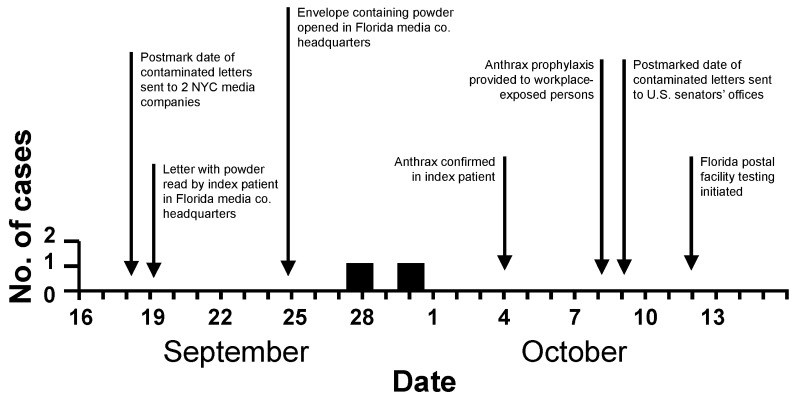
Dates of onset of symptoms of inhalational anthrax cases in Florida, and timeline of related events, September 16–October 16, 2001.

Workplace information about exposure to suspicious mail indicated that the incubation period for both Florida case-patients was <12 days (Figure 1). The index patient had onset of illness 11 days after handling suspicious mail on September 19. The second case-patient had illness onset September 28, 9 days after the index-patient viewed suspicious mail on September 19 and 3 days after his co-worker opened a letter with powder in it on September 25.

Serial or paired serum tests for IgG antibody response to the PA component of the anthrax toxins were performed on serum of 436 workplace-exposed persons. No serum indicated a reaction consistent with acute B. anthracis infection except for that of the second case-patient. For most of the serologic tests, specimens were collected on October 10 and 17.

Among 32 postal workers who potentially handled workplace mail at two county postal facilities, 31 nasal cultures were obtained; none yielded B. anthracis. No anthrax cases were detected among 3,263 postal workers working at the 51 Palm Beach County postal facilities through the county postal worker surveillance system, which reported 226 illnesses and 7 hospitalizations during October 25–November 9, 2001.

### Environmental Investigation

Of 136 investigation-directed environmental samples obtained during October 8–10 from the workplace and company mail van, 20 were positive, including 10 of 20 from the mailroom, 1 of 2 from the company mail van, 5 of 6 from the office of the asymptomatic mail-sorter who had a positive nasal culture and had opened a letter containing powder, 2 of 21 from the index patient’s work area (at an incoming-mail desk near his workspace and a repeat sample from his computer keyboard), 1 of 9 in the text library, and 1 from the single basement ventilation filter sample. No B. anthracis contamination was detected from 8 trash receptacles or 2 roof ventilation filters, 28 bulk items removed from the building containing security camera information, 18 samples from a construction area, or 21 other samples from other work areas and the entrance lobby. Five samples from the third-floor HVAC ducts (three from the index patient’s office and one from another office), and three samples from the first floor HVAC ducts (from the mailroom, an office where an envelope with powder was opened, and the text library) were negative.

**Figure 2:**
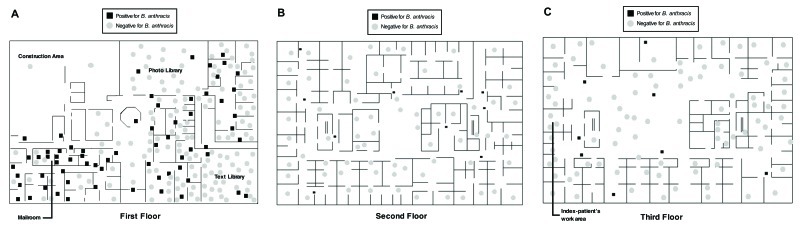
Environmental sample locations of specimens tested for Bacillus anthracis obtained October 25–November 8, 2001, on the three floors of the media company building where patients were employed, Palm Beach County, Florida. Sample locations of 59 negative specimens (including 46 air samples) are not depicted.

Eighty-four of 460 workplace samples obtained during October 25–November 8 yielded B. anthracis (Figure 2). Isolates of B. anthracis were obtained from 66 of 247, 10 of 95, and 8 of 112 samples from the first, second, and third floors, respectively; none of 6 specimens were positive from the parking garage or roof vents. The northeast quadrant of the third floor, which contained executive office suites, a conference room, and storage areas, was the only quadrant of any floor without detected contamination. The index-patient’s office was located on the third floor of the building. The mailroom (the work area of the second case-patient) and the office near the mailroom where a powder-containing letter was opened are both on the first floor.

No mail containing B. anthracis spores was recovered. Because workplace refuse is incinerated and waste receptacles did not show contamination, no environmental specimens were obtained from waste sites.

B. anthracis contamination was detected at six of seven postal facilities tested, from routes serving the current workplace headquarters and a former office. Contamination was not detected at a facility that receives mail addressed to the post office box of the former workplace office, last used 13 months earlier.

Molecular subtyping analysis (MLVA) was performed on one B. anthracis isolate recovered from a postal facility that processed workplace mail, 18 isolates recovered from the workplace, cerebrospinal fluid and blood culture isolates from the index patient, and two nasal swab isolates from workplace-exposed persons. All B. anthracis isolates tested were indistinguishable by MLVA.

### Prophylaxis and Control Measures

Beginning October 8, we recommended 60-day antibiotic postexposure prophylaxis (2) to 1,114 workplace-exposed persons identified through employers and responses to public service announcements. We provided medication refills on October 17–19 and November 1 at a workplace branch office and as needed through the Palm Beach County Health Department. Beginning October 24, we attempted telephone contact with persons who did not refill medications and advised them about our recommendations and how to obtain medications. Adjunct anthrax vaccination, available beginning December 22, was accepted by three workplace-exposed persons.

When the postal system risk assessment was initiated on October 12, antibiotic prophylaxis was offered to 32 postal workers who were most likely to have handled workplace mail at two local postal facilities. After we determined that at least 24 days had passed since contamination most likely took place in postal facilities, we did not recommend prophylactic antibiotics to Florida postal workers since more than two of the typical 1- to 7-day incubation periods for inhalational anthrax had passed, or two of the up to 12-day incubation periods estimated for the two Florida cases.

## Discussion

This report describes the investigation of the first bioterrorism-related anthrax case identified in the United States. We detected two inhalational anthrax cases (including the index case) among workers of a Florida media company. Anthrax transmission and widespread environmental contamination throughout the workplace and in six local postal facilities most likely resulted from two letters containing B. anthracis spores delivered to the workplace.

The index patient’s infection most likely occurred from inhalation of B. anthracis spores following a primary aerosolization, i.e., spores released into the air after opening a spore-containing letter. This scenario is consistent with co-workers’ recollections that the index patient held a letter containing powder over his computer keyboard, as well as environmental samples showing contamination at his keyboard, an incoming-mail desk near his workspace, and his mailroom mailbox. The second case-patient did not recall opening or seeing a letter containing powder, and the mechanism of spore aerosolization resulting in his infection is unclear. He was likely exposed while delivering 10,000–15,000 mail pieces daily to the workplace mailroom; both the mailroom and mail van were contaminated with B. anthracis spores. He may have inhaled spores after mail was compressed or shaken during delivery or after he (unknowingly) or a co-worker opened a spore-containing envelope. A secondary aerosolization, i.e., spores resuspended in the air after settling to a surface following an initial release, may also have resulted in his infection.

Results from environmental specimens and nasal swab cultures helped guide the investigation and were especially useful when combined as epidemiologic tools. The first environmental sample yielding B. anthracis, from the index patient’s work area, when paired with the first positive nasal swab culture, which was obtained from the second case-patient, indicated that the exposure source was at the workplace. Evidence that mail was the transmission vehicle was provided through two nasal swab cultures yielding B. anthracis from workplace mail handlers (one who recalled opening a letter containing powder) and results of environmental specimen cultures, revealing contamination in the workplace mail van and mail room. The usefulness of nasal swab cultures may have been limited by the interval of >13 days between the primary aerosolized spore exposures (letters opened on or about September 19 and 25) and the date nasal cultures were obtained (most on October 8). A high yield from nasal cultures would not be expected after >7 days had elapsed. One study showed that only one of eight nasal cultures from rhesus monkeys exposed to aerosolized B. anthracis spores yielded B. anthracis 7 days later (11). Environmental sampling was valuable independently in areas where no contamination was detected, by directing the investigation away from uncontaminated areas.

Environmental sampling revealed widespread contamination. However, the number or percentage of positive samples in a given area could not be used to quantify the contamination because quantitative spore counts were not performed when samples were cultured, a variety of sampling techniques were used (swabs, wipes, vacuum, and air sampling), and the distribution of samples obtained was not uniform.

This report documents the public health investigation into the first recognized case of anthrax due to intentional dissemination of B. anthracis spores in the United States. We demonstrated the usefulness of nasal swab cultures when combined with environmental specimen and epidemiologic data to identify the exposure site and vehicle used for anthrax transmission. Public health workers and clinicians should remain vigilant for anthrax because of the continued threat of bioterrorism.
